# Cell surface binding, uptaking and anticancer activity of L-K6, a lysine/leucine-rich peptide, on human breast cancer MCF-7 cells

**DOI:** 10.1038/s41598-017-08963-2

**Published:** 2017-08-15

**Authors:** Che Wang, Shaodan Dong, Lin Zhang, Ying Zhao, Lili Huang, Xiange Gong, He Wang, Dejing Shang

**Affiliations:** 1 Department of Pharmacy, School of Chemistry and Chemical Engineering, Liaoning Normal University, Dalian, 116029 China; 2 Liaoning Provincial Key Laboratory of Biotechnology and Drug Discovery, School of Life Sciences, Liaoning Normal University, Dalian, 116081 China

## Abstract

Cell surface binding and internalization are critical for the specific targeting and biofunctions of some cationic antimicrobial peptides (CAPs) with anticancer activities. However, the detailed cellular process for CAPs interacting with cancer cells and the exact molecular basis for their anticancer effects are still far from being fully understood. In the present study, we examined the cell surface binding, uptaking and anti-cancer activity of L-K6, a lysine/leucine-rich CAP, in human MCF-7 breast cancer cells. We found that L-K6 preferentially interact with MCF-7 cells. This tumor-targeting property of L-K6 might be partially due to its interactions with the surface exposed and negatively charged phosphatidylserine. Subsequently, L-K6 could internalize into MCF-7 cells mainly through a clathrin-independent macropinocytosis, without significant cell surface disruption. Finally, the internalized L-K6 induced a dramatic nuclear damage and MCF-7 cell death, without significant cytoskeleton disruption and mitochondrial impairment. This cytotoxicity of L-K6 against MCF-7 cancer cells could be further confirmed by using a mouse xenograft model. In summary, all these findings outlined the cellular process and cytotoxicity of L-K6 in MCF-7 cancer cells, and might help understand the complicated interactions between CAPs and cancer cells.

## Introduction

Cancer is a major public health problem worldwide and is the second leading cause of death in the United States^1^. Chemotherapy or biochemotherapy is currently the standard treatment strategy for various cancers. However, the development of cancer resistance and specific tumor microenvironment lead to the insufficient selectivity for cancerous rather than normal cells. Consequently, potential toxicity and many other significant side effects greatly restrict the clinical applications of those conventional therapies. Hence, the design and development of novel anticancer drugs preferentially targeting cancer cells is an important endeavor in anticancer research.

Recently, various peptide sequences, from either natural or synthesized sources, have been reported to selectively interact with specific molecular markers, receptors or other tumor cell components, and have been of value for application in cancer treatment^2^. For instance, many cationic antimicrobial peptides (CAPs) or cell penetrating peptides (CPPs) are found to exhibit specific cytotoxicities against a broad spectrum of human cancer cells both *in vitro* and *in vivo* ^3–10^, via pore formation (cell membrane lysis) and/or non-pore dependent intracellular mechanisms^11–15^. However, the exact molecular and cellular process of these peptides in cancer cells, including binding, uptaking and biofunctioning, are still far from being fully understood due to the complicated physical and chemical properties of peptides and great variety of organelles and structures/micro-structures of different cancer cells.

We recently designed a series of cationic peptidic analogues of temporin-1CEb, a natural CAP isolated from the skin secretions of Chinese brown frog (*Rana chesinensis*)^16–18^. In the present study, we found that these peptidic analogues (as summarized in Table 1) showed preferential cytotoxicities against various cancer cells, with MCF-7 breast cancer cells the most sensitive. In addition, among these peptidic analogues, L-K6 possessed the strongest anticancer activity with relatively lower cytotoxicity against non-cancerous HaCaT cells. Therefore, in the present study, we investigated the cellular processes of L-K6 in MCF-7 cells, including its cell surface binding, cellular uptaking and its impacts on intracellular constituents. We found that the negatively charged phosphatidylserine (PS), which has been reported to be abundantly exposed on cancer cells surface^19, 20^, might contribute to the preferential binding of L-K6 with MCF-7 cancer cells. Additionally, after interacting with cancer cells surface, L-K6 peptide internalized into MCF-7 cells mainly via a clathrin-independent macropinocytosis,  without significant cell surface disruption. Moreover, the internalized L-K6 caused dramatic nucleus damage in MCF-7 cells, without significant cytoskeleton and mitochondria disruption, indicating a nucleus targeting capacity and a promising potential to serve as a potential candidate for a novel peptidic anticancer drug.

**Table 1:** Amino acid sequences and physical characteristics of L-K6 and its analogues.

Peptides	Amino acid sequence	MW	Net charge	Mean hydrophobicity^a^
L-K6	IKKILSKIKKLLK-NH_2_	1,552.1	+6	11.6
L-K5 V1	IKKIVSKIKKLL-NH_2_	1,410.0	+5	11.5
L-K6 V1	IKKIVSKIKKLLK-NH_2_	1,538.1	+6	10.9

^a^The mean hydrophobicity values of the peptides were calculated using the hydrophobicity scales and were represented as the total hydrophobicity (sum of all residue hydrophobicity indices) divided by the number of residues. All residue hydrophobicity indices were defined as follows: Trp, 32.4; Phe, 29.1; Leu, 23.3; Ile, 21.4; Met, 15.7; Tyr, 14.7; Val, 13.4; Pro, 9.0; Cys, 7.6; Lys, 2.8; Glu, 2.8; Ala, 2.8; Thr, 2.3; Asp, 1.6; Arg, 0.6; Gln, 0.6; His, 0; Ser, 0; Gly, 0; and Asn, −0.6.

## Results

### Specific cytotoxicity of the lysine/leucine-rich peptide L-K6 against cancer cells

**Figure 1:**
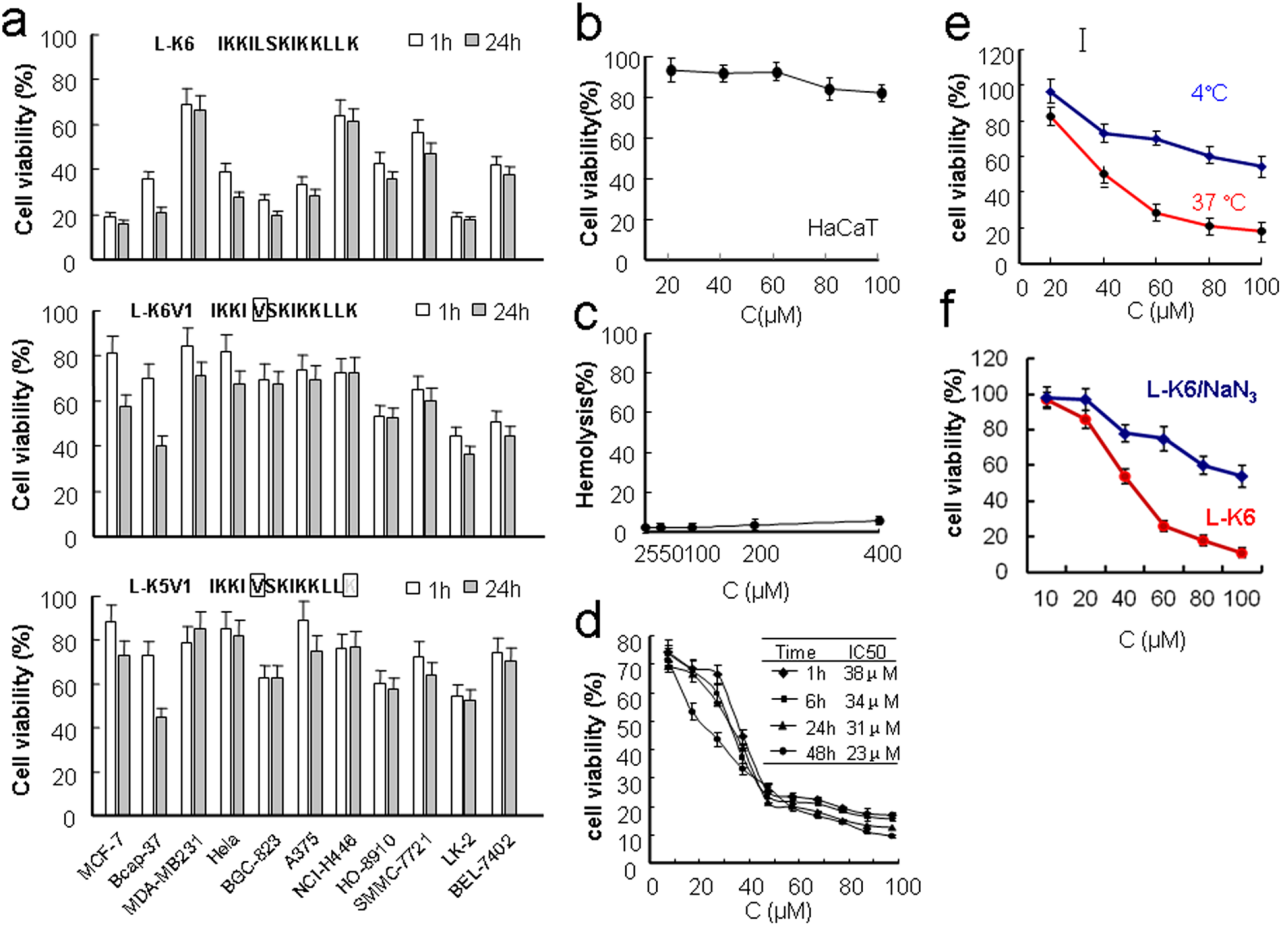
Cytotoxicity of lysine/leucine-rich peptides as assessed by MTT assay. L-K6 exerts much stronger cytotoxicity against various cancer cell lines than L-K6V1 and L-K5V1, with MCF-7 cells exhibiting the most sensitivity (**a**). In contrast, L-K6 exerts much less cytotoxicity against non-cancerous HaCaT cells (**b**). The hemolysis assay further supported the cancer-targeting property of L-K6 (**c**). Additionally, MTT assay indicated that L-K6 rapidly reduced the viability of MCF-7 cells in a dose- and time-dependent manner (**d**). Interestingly, our data also revealed that the cytotoxicity of L-K6 on the MCF-7 cells was thermo-sensitive (**e**) and energy-dependent (**f**). All experiments had been performed in triplicate. The data were expressed as mean ± SD.

The cytotoxicity of L-K6 on cancer cells was first determined using the MTT assay. As shown in Fig. 1a, L-K6 exerted significant cytotoxicity on various cancer cell lines, with MCF-7 cells exhibiting the highest sensitivity. In addition, substitution of the leucine residues (L-K6V1) and deletion of the lysine residues (L-K5V1) in the peptide dramatically weakened this cytotoxicity.

To evaluate the cancer specificity of L-K6-induced cytotoxicity, non-cancerous HaCaT cells were treated with various concentrations of L-K6 and the cells viability was determined by MTT assay. The data clearly indicated that L-K6 exerted minimum toxicity against normal HaCaT cells after 1-hour peptide exposure (Fig. 1b). Moreover, the hemolysis assay also consistently supported the cancer-targeting property of L-K6 (Fig. 1c).

Because MCF-7 cell line is most sensitive to L-K6, the cytotoxic properties and cellular process of L-K6 were further evaluated using this cell line. The data from MTT assay further indicated that L-K6 rapidly reduced the viability of the MCF-7 cells in time- and dose-dependent manners, with IC_50_ values as 38 μM, 34 μM, 31 μM and 23 μM after 1 hour, 6 h, 24 h and 48 h peptides exposure, respectively (Fig. 1d).

Moreover, when MCF-7 cells were treated with L-K6 (20–100 µM) at 4 °C for 1 hour, we found that low temperature ameliorated the cytotoxicity of L-K6 (Fig. 1e), indicating a thermo-sensitive and energy-dependent property of L-K6-induced cytotoxicity in MCF-7 cells. Additionally, the energy-dependent cytotoxicity of L-K6 was further confirmed when the cellular ATP pool was depleted by pre-incubating the cells with sodium azide (NaN_3_) (Fig. 1f).

### L-K6 induces slight membrane permeabilization specifically in MCF-7 cells

**Figure 2:**
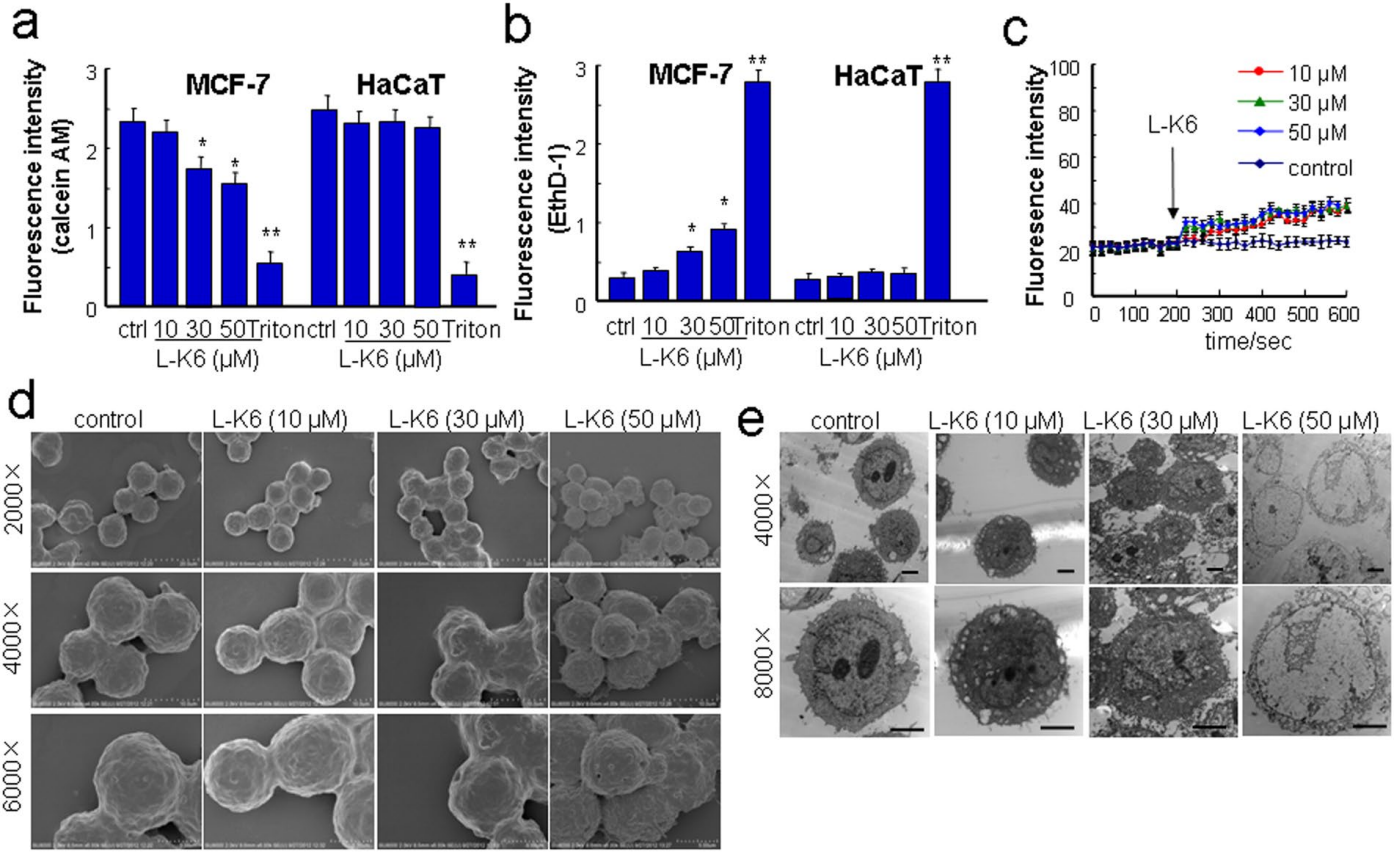
L-K6 at higher concentrations preferentially elevated cancer cell membrane permeability. To evaluate the possible impacts of L-K6 on cell membrane permeability, a calcein/EthD-1 leakage assay was performed. L-K6 administration (especially 30~50 μM) induced a significant change in membrane permeability of MCF-7 cells, as indicated by the decreased fluorescence intensities of calcein (**a**) and increased EthD-1 (**b**). Additionally, L-K6-induced elevation of membrane permeability was cancer-specific, as evidenced by stable fluorescence intensities of calcein and EthD-1 in HaCaT cells (**a**,**b**). Consistent with calcein/EthD-1 leakage assay, the membrane depolarization assay further supported the elevated membrane permeability (mean ± SD) as indicated by the increased DiBAC_4_(3) fluorescence intensity after peptides exposure (**c**). Morphological changes of human MCF-7 breast cancer cells induced by L-K6 as assessed by scanning electron microscopy (**d**) and transmission electron microscopy (**e**). *p < 0.05, **p < 0.01 vs. untreated control group. All experiments had been performed in triplicate.

L-K6 exposure induced calcein AM/EthD-1 leakages in MCF-7 cells, as indicated by the decreased fluorescence intensity of calcein and the increased fluorescent intensity of EthD-1 (Fig. 2a and b). In contrast to MCF-7 cells, HaCaT cells were relatively resistant to L-K6 (Fig. 2a and b), suggesting a cancer cell-specificity of L-K6-induced membrane permeabilization. The membrane depolarization assay further confirmed the slight membrane permeabilization induced by L-K6, as shown by a slight elevation of transmembrane potential (Fig. 2c).

Moreover, while L-K6 induced slight membrane permeabilization, the electronic microscope observations revealed a relatively intact cell surface (by scanning electronic microscopy, Fig. 2d) and a dramatic nuclear damage (by transmission electronic microscopy, Fig. 2e) in L-K6-treated MCF-7 cells, especially at 50 μM.

### L-K6 induces morphological changes of membrane preferentially in MCF-7 cells

**Figure 3:**
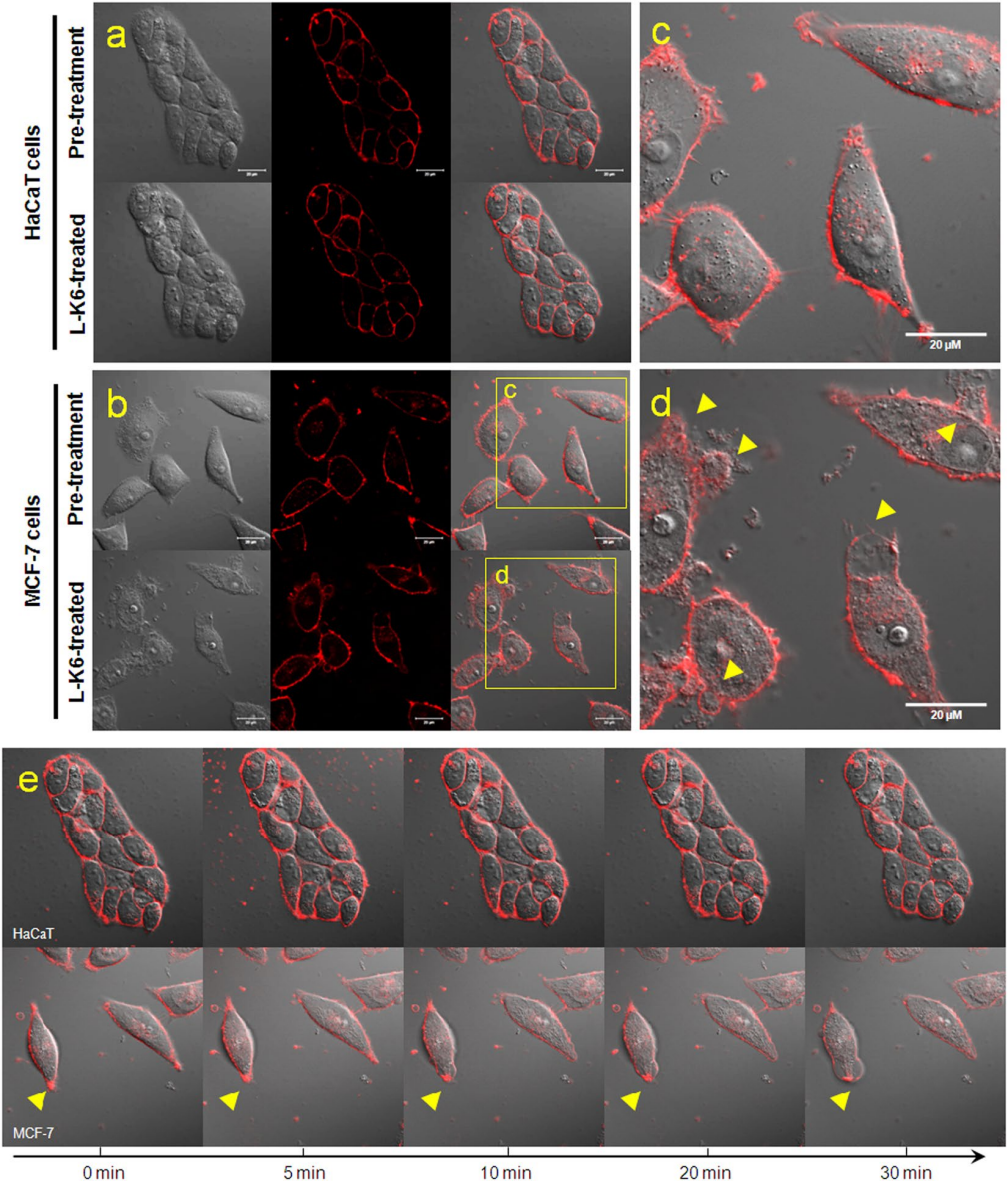
Morphological changes of the HaCaT or MCF-7 cell membranes after L-K6 treatment. The HaCaT (**a**) or MCF-7 (**b**) cells were cultured and treated with 50 μM L-K6 for 30 min. The membrane fluorescence image indicated that while the HaCaT cell membrane showed no significant morphological changes following 30 min of L-K6 exposure, dramatic morphological changes were observed in the MCF-7 cell membrane, as indicated by cytoplasmic membrane perturbations and blebbing [yellow arrowheads; (**c**) pre-treatment; (**d**) post-treatment]. In addition, the dynamic tracing indicated that these morphological changes occurred as early as 10 min after L-K6 exposure (**e**).

Consistent with calcein AM/EthD-1 staining assay and electronic microscope observation, the membrane fluorescence imaging indicated that the non-cancerous HaCaT cells showed no significant morphological changes (Fig. 3a) after 30 min exposure with 50 μM L-K6. In contrast, 50 μM L-K6 exposure induced significant morphological changes in MCF-7 cells membrane, as shown by cytoplasmic membrane perturbations and blebbings (yellow arrowhead Fig. 3b–d), suggesting an elevated permeability of the MCF-7 cell membrane. In addition, the live-cell imaging indicated that these morphological changes of MCF-7 cells occurred as early as 5–10 min after L-K6 exposure (Fig. 3e).

### L-K6 specifically internalizes into MCF-7 cells in a dose-dependent manner

**Figure 4:**
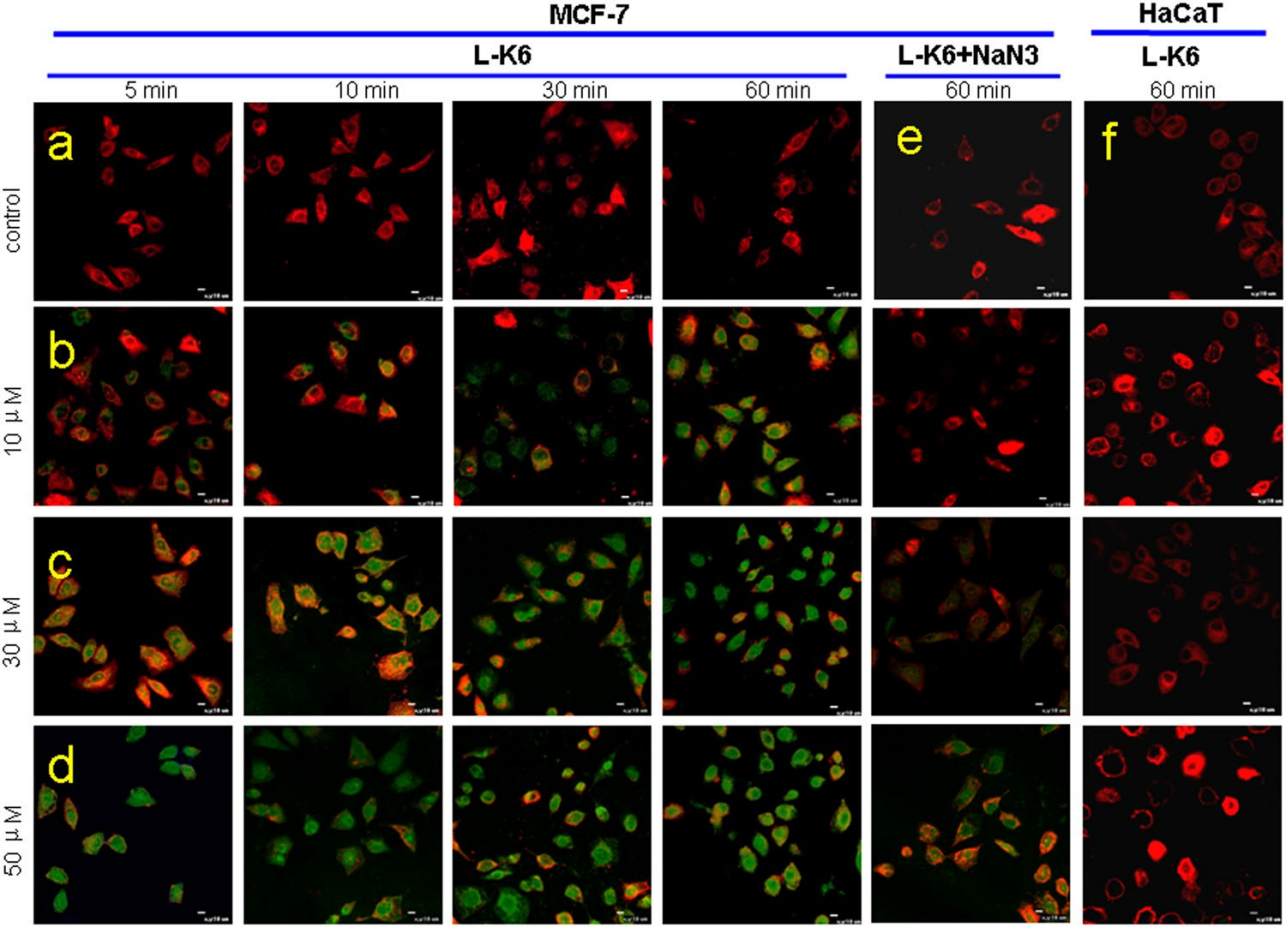
L-K6 specifically enters MCF-7 cells in a dose-dependent manner. At the beginning of the observation, lower concentrations (10 μM) FITC-labeled L-K6 initially accumulated around the tumor cell membranes. However, with increasing peptide concentrations and incubation times, the location of the FITC-labeled L-K6 was transferred into the cytoplasm, as indicated by the increased intracellular green fluorescence intensity (**a**–**d**). Moreover, pre-incubation of MCF-7 cells with NaN_3_ blocked the uptake of L-K6 (**e**). In contrast to MCF-7 cells, HaCaT cells displayed negligible cellular uptake levels of L-K6 even after 60 min exposure (**f**).

After incubation with the MCF-7 cells, the cellular localization of L-K6 was traced by laser confocal microscopy at different time points. As shown in Fig. 4a–d, L-K6 can rapidly internalize into cells, as shown by the intracellular green fluorescence, after 5 min of peptides exposure. Moreover, pre-incubation of MCF-7 cells with 40 μM NaN_3_ inhibited the cellular uptake of L-K6 (Fig. 4e), suggesting an energy-dependent transportation of L-K6 across MCF-7 cell membrane.

In contrast to MCF-7 cells, non-cancerous HaCaT cells displayed a negligible cellular uptake of L-K6 even after 1-hour exposure (Fig. 4f), indicating a cancer-targeting uptake of L-K6.

### Effects of endocytosis inhibitors on the uptake of L-K6 by MCF-7 cells

**Figure 5:**
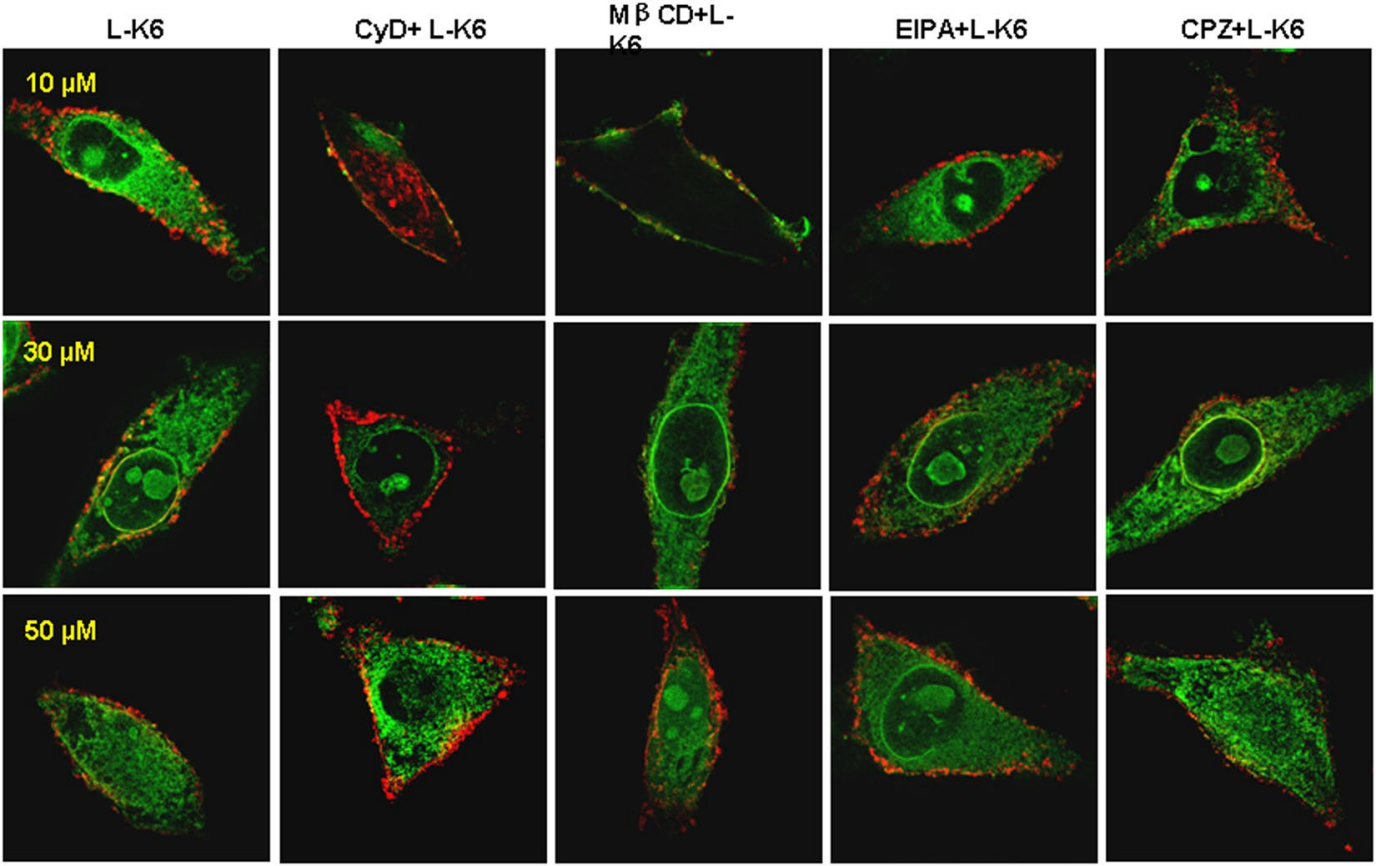
Cellular uptake of L-K6 by MCF-7 cells was partially blocked by endocytosis inhibitors, as assessed by 3D-SIM super-resolution microscopy. Pre-incubation with cytochalasin D (CyD) dramatically blocked the internalization of L-K6 at 10–30 μM. Similar inhibitory effect was observed in cells pretreated with methyl-β-cyclodextrin (Mβ-CD), a membrane cholesterol depleting agent and caveolae-mediated endocytosis inhibitor. Interestingly, this effect was much more sensitive to 10–30 μM L-K6. Additionally, ethylisopropylamiloride (EIPA) significantly decreased fluorescence derived from FITC-L-K6 (particularly at 50 μM). In contrast, chlorpromazine (CPZ), a clathrin-dependent endocytosis inhibitor, showed no effect on cellular uptake of L-K6.

The data from MTT assay and confocal microscope observation suggested an energy-dependent manner of L-K6 cellular uptake. Therefore, by using various endocytosis inhibitors, we next investigated whether endocytic pathways were involved in the cellular uptake of L-K6 by MCF-7 cells. MCF-7 cells were treated with FITC-labeled L-K6 (10–50 μM) in the presence or absence of various endocytosis inhibitors for one hour. The cellular uptake of peptides was determined by using super-resolution microscopy, FACS analysis or microplate reader. As shown in Fig. 5 and Suppl Fig. S1a, methyl-β-cyclodextrin (Mβ-CD), a caveolae-mediated endocytosis inhibitor, reduced the cellular uptake of L-K6. Another macropinocytosis inhibitor, cytochalasin D (CyD), also significantly blocked the internalization of L-K6, as evidenced by an over 90% reduction of L-K6 at 10–30 μM and an approximately 50% reduction at 50 μM (Fig. 5 and Suppl Fig. S1a). In contrast, macropinocytosis inhibitor ethylisopropylamiloride (EIPA) decreased the cellular uptake of L-K6 only at 50 μM (Suppl Fig. S1a). Additionally, chlorpromazine (CPZ), a inhibitor of clathrin-mediated endocytosis, showed no ameliorating potential against the internalization of L-K6 into MCF-7 cells (Suppl Fig. S1a). All these data suggested that a clathrin-independent macropinocytosis pathway was involved in the cellular uptake of L-K6 by MCF-7 cells. The microplate reader assay revealed similar impacts of endocytosis inhibitors on the cellular uptake of L-K6 (Suppl Fig. S1b, left panel). Moreover, the microplate reader assay also indicated that the cellular uptake of L-K6 was blocked by low temperature (4 °C) (Suppl Fig. S1b, right panel), suggesting a thermo-sensitive and energy-dependent fashion of L-K6 cellular uptake.

To further explore the impacts of various endocytosis inhibitors on L-K6-induced cytotoxicity, the MCF-7 cells were treated with L-K6 in the presence of endocytosis inhibitors Mβ-CD, CyD, EIPA or CPZ for one hour. The cellular viability was determined by using MTT assay. The data clearly revealed that endocytosis inhibitors, with the exception of CPZ, partially ameliorated the cytotoxicity of L-K6 against the MCF-7 cells (Suppl Fig. S1c), as evidenced by increased IC_50_ values (Suppl Fig. S1c,d).

### L-K6 elevates membrane permeability partially via a PS-related mechanism

**Figure 6:**
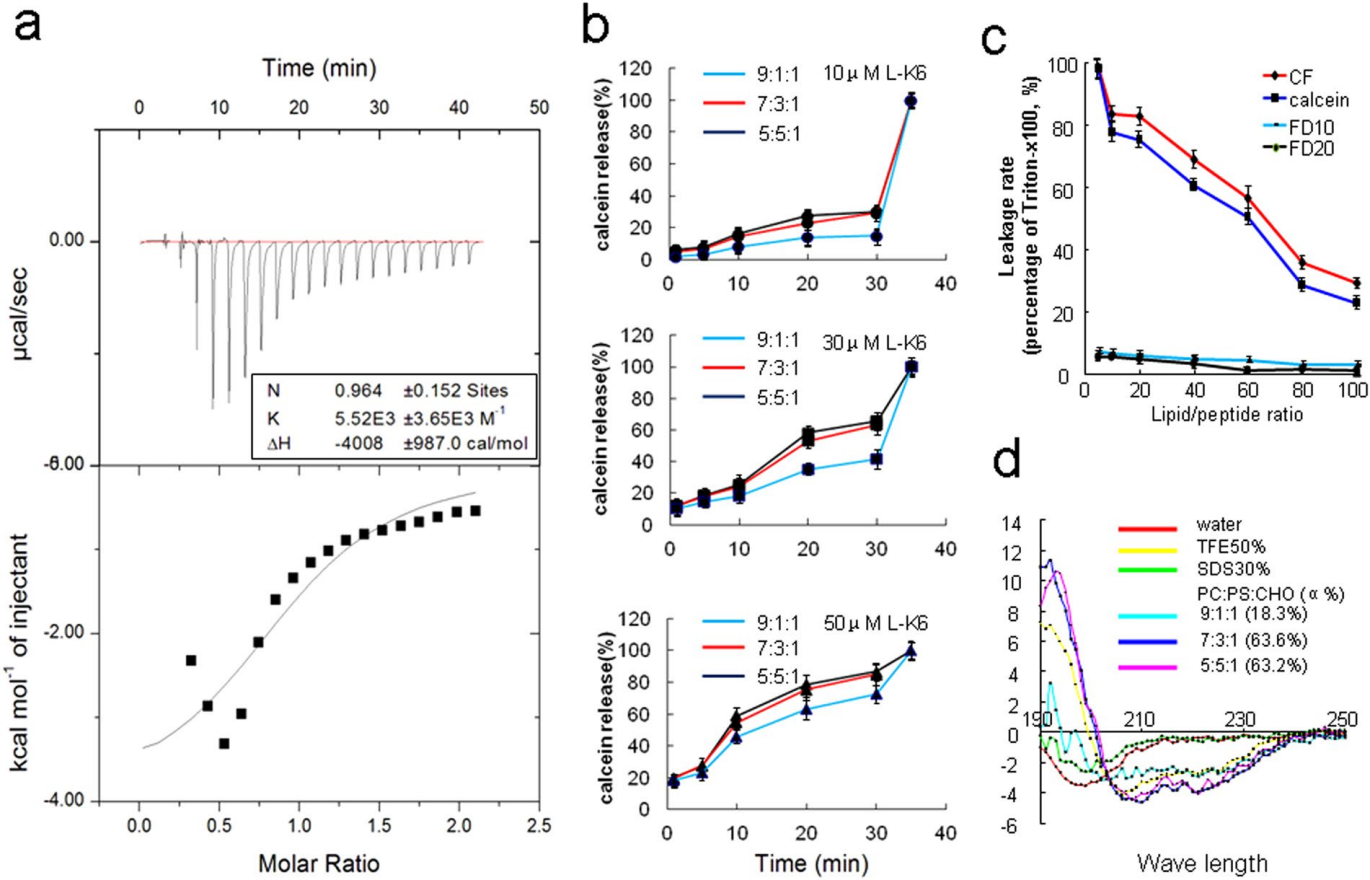
L-K6 elevated the liposome permeability through a PS-related mechanism. The ITC data suggested the direct binding of the positively charged L-K6 to the negatively charged PS (**a**). Consistent with the data from the cell-based system, co-incubation of L-K6 with the liposomes caused rapid leakage of calcein from the liposomes in a dose-dependent manner. In addition, this leakage activity was PS-associated, as shown by an elevated calcein intensity with increased PS content of liposome (from 9:1:1 to 7:3:1 or 5:5:1) (**b**). Interestingly, calcein and carboxyfluorescein (CF) were released from the liposome, whereas FD10 and FD20, two fluorescent dyes with a molecular weight over 10kD, were resistant to be released (**c**). Moreover, the higher ratio of PS content in liposome yielded a higher percentage of α-helical structure, as assessed by circular dichroism spectroscopy (**d**). All experiments had been performed in triplicate. All data were expressed as mean ± SD.

The interactions between L-K6 and PS were first evaluated using ITC assay. While no signal was observed in control test with PBS injection into L-K6 peptides (almost no heat exchanges was developed for each injection, Suppl Fig. S3), the data suggested that the positively charged L-K6 could directly bind to negatively charged PS (Fig. 6a). However, the trend line is not good fitted suggesting a complex interaction between PS and l-K6. The titration curve was elevated rapidly and then fell back slowly. This two-mode of binding observed here might be explained by an aggregation of peptide to reach a required concentration for the ITC experiment and in the presence of high local concentrations of liposomes after each addition. Moreover, the cholesterol containing in the liposomes may also have potential impacts on PS-peptides interactions.

To further evaluate the potential contributions of PS in L-K6-induced membrane permeability, the liposomes encapsulated calcein was prepared and the fluorescence dye leakage was determined using a membrane permeabilizing activity assay. Consistent with the data from cell-based system, co-incubation of L-K6 with the liposomes caused a rapid leakage of calcein from liposomes in a dose- and time-dependent manner (Fig. 6b). In addition, this activity was closely associated with the PS concentration, as shown by the elevated calcein release from liposomes following the increased PS ratio [from 9:1:1 to 7:3:1 or 5:5:1, phosphatidylcholine (PC)/PS/cholesterol (CHO) (mol/mol)] (Fig. 6b). Interestingly, while calcein (molecular weight 622.6 Da) and carboxyfluorescein (CF, molecular weight 376.3 Da) were released out of the liposomes with the presence of L-K6, FD10 and FD20, two fluorescent dyes with larger molecular weights (over 10 kD and 20 kD, respectively), were resistant to be released (Fig. 6c). These data suggested that L-K6 could slightly elevate membrane permeability to some extent but not sufficient to cause membrane disruption. Moreover, the circular dichroism (CD) spectra analysis indicated that a higher ratio of PS content in the liposomes yielded a higher percentage of α-helical structure of peptides (Fig. 6d), which may also faciliate the internalization of L-K6 into the MCF-7 cells.

### The nuclear-targeting and DNA disrupting activities of L-K6

**Figure 7:**
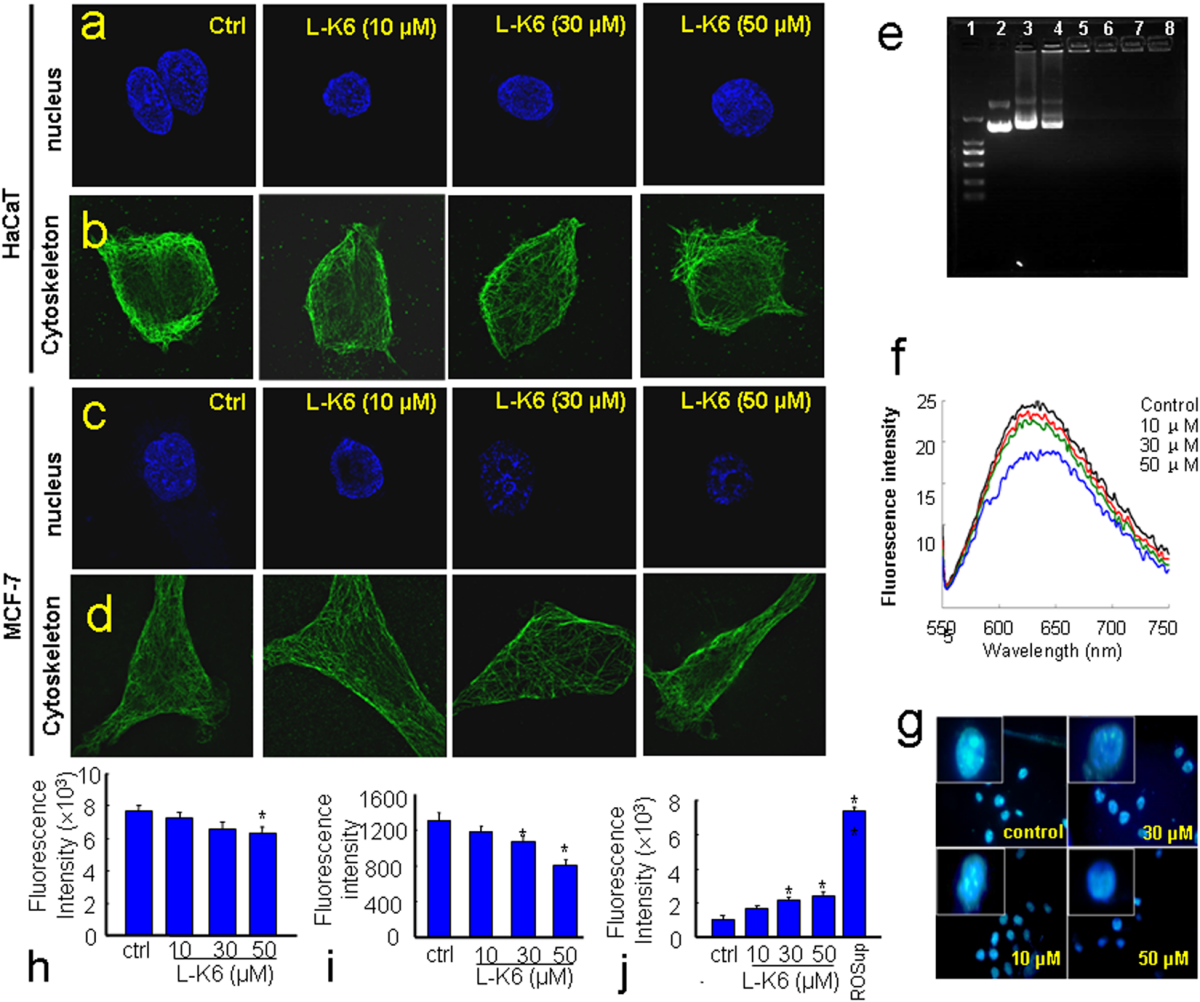
L-K6-induced nuclear damage in MCF-7 cells. The data from super-resolution microscopy observation clearly revealed that the cytoskeleton and nucleus of the HaCaT cells remained intact after L-K6 exposure (**a**,**b**). In contrast, L-K6 exposure induced significant nuclear damage in the MCF-7 cells, as indicated by the diminished blue fluorescence (**c**), without significant interruption of the cytoskeleton (**d**). Moreover, the DNA binding activity of L-K6 was evaluated using an electrophoretic gel mobility shift assay. (1) λ-Hind III digested DNA Marker; (2–8) the peptide:DNA mass ratios are 0:1, 0.5:1, 1:1, 5:1, 10:1, 50:1 and 100:1, respectively (**e**). The fluorescence spectra were measured from 550 to 750 nm (Ex 535 nm). When the concentrations of L-K6 increased, the fluorescence of the EB–DNA system decreased (**f**). A one-hour treatment with L-K6 consistently resulted in a dose-dependent reduction of DAPI fluorescence brightness in the MCF-7 cells (**g**), which may indicate chromatin decondensation and DNA double strands breaks. FACS analysis was performed to detect the mitochondrial membrane potential, intracellular calcium (Ca^2+^) levels and ROS production, after a one-hour L-K6 exposure to MCF-7 cells. The data indicated that after one hour of treatment, L-K6 induced only a slight reduction of the mitochondrial membrane potential (**h**). The cytosolic Ca^2+^ level was also slightly decreased at higher concentrations (**i**). In addition, ROS production was also elevated after one hour of L-K6 exposure (**j**). *p < 0.05, **p < 0.01 vs. untreated control group. All experiments had been performed in triplicate.

The MCF-7 or HaCaT cells were co-incubated with L-K6 (10, 30 and 50 μM) for 1 hour and the cytoskeleton or nucleus were labeled with anti-α-tubulin/Alexa Fluor 488 or Hoechst33342. Super-resolution microscopy observation clearly revealed that both cytoskeleton and nucleus of HaCaT cells remained intact after L-K6 exposure (Fig. 7a and b). In contrast, one-hour exposure with L-K6 caused a dramatic nuclear damage in MCF-7 cells, as shown by the diminished blue fluorescence (Fig. 7c). Interestingly, one-hour L-K6 exposure caused no significant cytoskeleton disruption in MCF-7 cells, as evidenced by intact green fluorescence (Fig. 7d).

The DNA binding capability of L-K6 was further evaluated by using EMSA assay. The results showed that L-K6 possessed DNA binding affinity and limited the mobility of plasmid DNA (Fig. 7e). Additionally, one-hour of L-K6 exposure caused a dose-dependent decrease in the fluorescence of the EB–DNA system (Fig. 7f). Moreover, as shown in Fig. 7g, one hour treatment of MCF-7 cells with L-K6 resulted in a dose-dependent reduction of DAPI fluorescence brightness, indicating a chromatin decondensation and DNA double strand breaks.

### L-K6 promoted ROS production without alterations in mitochondrial membrane potential and intracellular Ca^2+^ levels

Since no significant membrane disruption was observed after L-K6 exposure, we then further explore the possible intracellular mechanisms underlying L-K6-induced cancer cell death. The mitochondrial membrane potential (MMP), intracellular Ca^2+^ levels and ROS production were determined by using FACs analysis. The data indicated that one-hour of L-K6 exposure induced slight decreases of MMP (Fig. 7h and Suppl Fig. S2a) and intracellular Ca^2+^ level (Fig. 7i and Suppl Fig. S2b). In contrast, the intracellular ROS production was significantly elevated after L-K6 exposure (Fig. 7j and Suppl Fig. S2c).

### Anticancer activity of L-K6 *in vivo*

**Figure 8:**
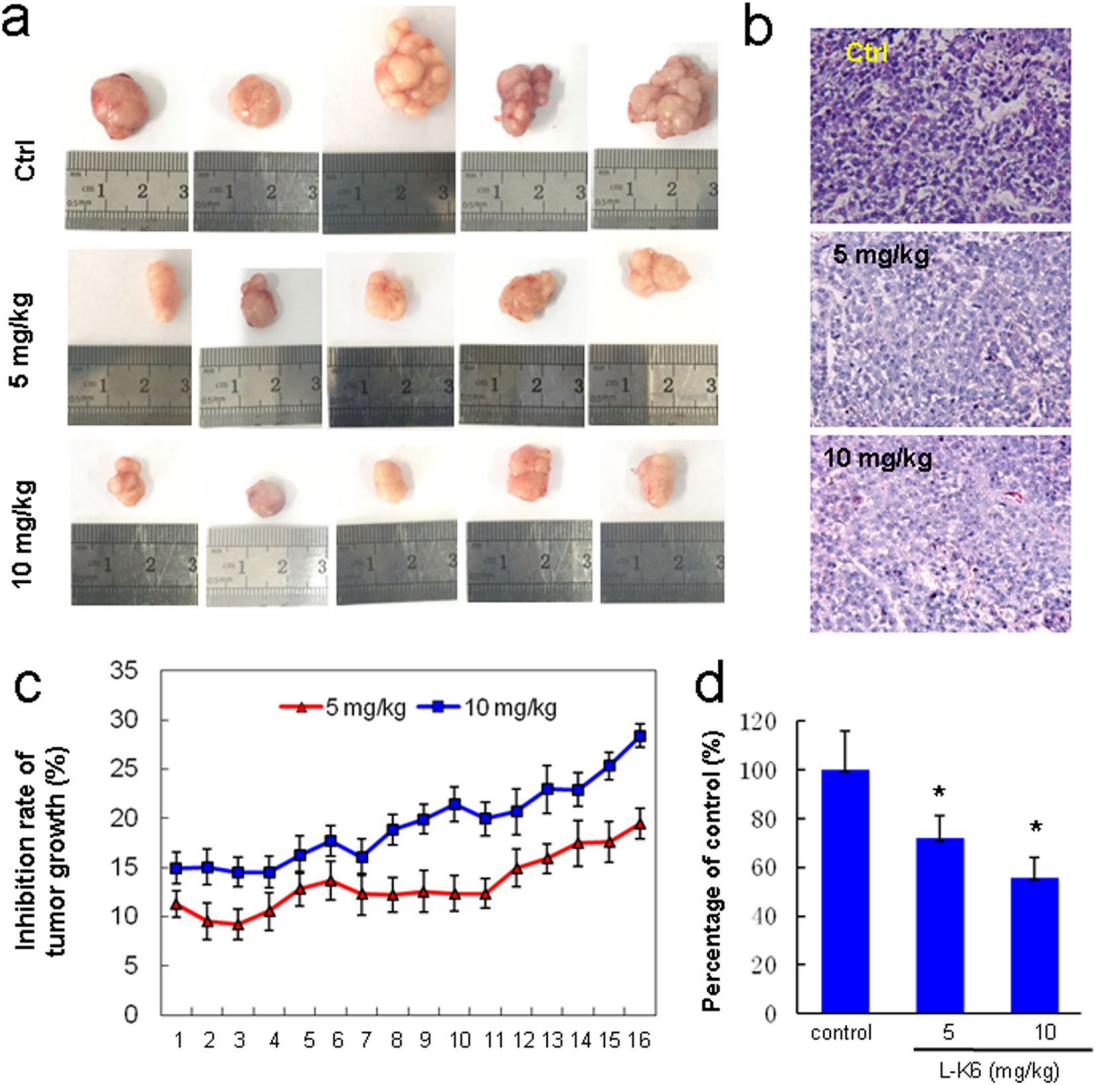
Anticancer activity of L-K6 *in vivo*. To further confirm the anticancer activity of L-K6 *in vivo*, nude mice bearing MCF-7 cancer were treated with L-K6 (5 mg/kg, 10 mg/kg) or NS as a control. The tumor size (mean ± SD) was measured everyday (**c**). At the end of the experiment, all animals were sacrificed and the tumor masses were weighed (**a**,**d**). The pathological analysis was performed by H&E staining (**b**). *p < 0.05 vs. untreated control group (n = 5).

To further confirm the *in vivo* anticancer activity of L-K6, nude mice bearing MCF-7 were locally treated with L-K6. After 15 days of L-K6 local injection, the mice were sacrificed, the tumors were carefully excised, and the cancer volume and weight were calculated. The data suggested that L-K6 inhibited the tumor growth, as shown by the reduced tumor size and weight (Fig. 8a,c and d).

To investigate the effect of L-K6 on the histology of MCF-7 xenografts, tumor sections taken from mice were stained with H&E. As shown in Fig. 8b, numerous cancer cells with higher nuclear fragmentation were apparent in H&E stained sections from L-K6-treated MCF-7 cells as compared to untreated control cells. All of the tumors are clearly carcinomas based on morphological characteristics.

## Discussion

Despite tremendous advances in current treatment modalities, cancer remains a major cause of mortality worldwide. Chemotherapy or biochemotherapy is currently one of the principal strategies for cancer treatment, particularly for patients with advanced or metastatic cancer^21^. However, the severe side effects in normal cells and tissues caused by the insufficient specificity against cancer cells are often obstacles for the clinical use of conventional therapeutics^22^. Thus, higher specificity to cancer cells, lower toxicity/side effects to the normal host cells, and a distinct mode of action represent new directions in the development of novel anticancer drugs. As one type of potential cancer therapeutics, some CAPs have been reported to show selective cytotoxicity and antitumor capabilities against a variety of cancer cell types. Previous studies have provided the modes of action for these CAPs, which involve the disruption or poration of the plasma membranes and other intracellular events^11, 23–26^. However, due to the complex bio-diversities of various cancer cells and the physicochemical properties of CAPs, the detailed cellular processes of these cancer-targeted CAPs, including the cancer cells surface binding and cellular uptaking, are still far from being clearly understood.

In the present study, we found that L-K6 preferentially induced cancer cell death with a relatively low cytotoxicity against non-cancerous HaCaT cells *in vitro*. Additionally, this anti-cancer activity could be further confirmed by using a mouse xenograft model. Cancer and normal mammalian cells have a number of differences that are accounted responsible for the selectivity of some CAPs. These differences rely firstly in the membrane net negative charge that characterizes malignant cells^7^. Anionic molecules such as PS, O-glycosylated mucins, sialylated gangliosides and heparin sulfate are present in the membrane of cancer cells, conferring them a net negative charge, which contrasts with the normal mammalian cell membrane. In fact, sialylated gangliosides and heparin sulfate have been indicated as potential targets for anticancer CAPs^27, 28^. Recently, PS on cancer cell surface has been demonstrated to be a critical target for some types of anticancer peptides or biomaterials and is also important for endocytosis^19, 29–32^. Our previous data revealed that MCF-7 cells surface expressed much higher level (over 10 folds) of PS than non-cancerous HaCaT cells and were much more sensitive to CAPs^20^. Consistent with these previous findings, in the present study, we found that L-K6 interacted preferentially with MCF-7 cancer cells and exerted selective anticancer activity, with relatively lower cytotoxicity to non-cancerous HaCaT cells. Moreover, the cancer-specific property of L-K6 might be partially mediated by a PS-related mechanism, which is supported by a direct binding of cationic L-K6 to anionic PS in ITC assay (Fig. 6a) and an elevated calcein leackage in L-K6-treated PS-containing liposome system (Fig. 6b). Interestingly, the data from CD spectra analysis in our present study further revealed that the negatively charged PS on cell surface not only attracted L-K6, but also promoted L-K6 to form α-helical structure, which has been reported to be critical for the membrane-translocation of peptidic molecules^33, 34^. Therefore, the preferential affinity of CAPs for specific components on lipid bilayers may be the key factor for their subsequent uptaking and cell-targeted cytotoxicity.

In the present study, after preferentially binding to cancer cell surface, L-K6 peptides were internalized into MCF-7 cancer cells and selectively killed cancer cells. The cellular mechanisms by which CAPs gain access into cancer cells are still under debate and far from being clear understood. Earlier studies suggested that most CAPs internalize into cells mainly through pore formation, which cause cell lysis, membrane permeabilization or other forms of bilayer disruption^35–37^. This conclusion was based on the observation that peptides enter the cells even at 4 °C, therefore, in an energy-independent manner. Besides this membrane poration-induced cell death, recent studies reported that the apoptosis and other intracellular events were also involved in CAPs’ cytotoxicity. For examples, BAA-2 caused mitochondrial-mediated apoptosis^24^, lower concentrations of gomesin and tachyplesin promoted apoptosis^25^.

In contrast to the energy-independent manner of those pore-forming peptides, our data indicated that the cellular uptake of L-K6 by cancer cells and the subsequent cytotoxicity were thermo-sensitive and energy-dependent (Figs 1e,f and 4e), suggesting endocytosis might be involved as a major uptake mechanism for L-K6. Endocytosis consists of several pathways including phagocytosis for the uptaking of large particles and pinocytosis for solute uptake. Pinocytosis is categorized as macropinocytosis, endocytosis dependent on the coat proteins clathrin or caveolin, or endocytosis independent of clathrin and/or caveolin^38^. Among them, clathrin or caveolin pits are involved in the mechanism of cellular uptake. Both clathrin and caveolin proteins, covering the intracellular part of the membrane, are required for invagination of membrane and help to form the vesicles after binding the extracellular molecule to the membrane. Our data found that the internalization of L-K6 could be partially blocked by caveolae-mediated endocytosis inhibitor Mβ-CD and macropinocytosis inhibitor CyD, but not by clathrin-mediated endocytosis inhibitor CPZ (Fig. 5 and Suppl Fig. S1), suggesting a clathrin-independent macropinocytosis involved in the internalization of L-K6 into MCF-7 cells.

Moreover, our data suggested that L-K6 interacted with cancer cells membrane and elevated the membrane permeability without significant membrane disruption. The electronic microscope observation revealed intact membrane morphology of MCF-7 cells after L-K6 treatment. In addition, the liposome-based assays further indicated that only small-sized molecules could be released after L-K6 exposure, suggesting an increased membrane permeability but not membrane disruption. These data further suggested that the cytotoxicity of L-K6 might be due to its intracellular biofunctions. After one hour exposure to MCF-7 cells, L-K6 caused only a slight mitochondrial membrane depolarization (Fig. 7h and Suppl Fig. S2a), and failed to cause cytoskeleton disruption (Fig. 7a–d). In addition, L-K6 distributed into cell nucleus, consequently disrupted cell nuclear (Fig. 7e–g), and ultimately resulted in cell death. Therefore, the anticancer mechanisms of L-K6 mostly rely on its nuclear damage and DNA disruption. Considering the enriched number of lysine and leucine residues in the sequence of L-K6, the present data may also provide a rational strategy for the design and development of anticancer drugs with effective and cancer-specific activities.

In conclusion, our findings revealed that L-K6 exerts anticancer activity both *in vivo* and *in vitro*. Additionally, L-K6 preferentially kills cancer cells with much lower toxicity to noncancerous cells via nucleus-targeting mechanism (Suppl Fig. S4). In detail, L-K6 specifically recognizes cancer cell surface partially via a PS-related mechanism, and crosses the MCF-7 cell membranes through a clathrin-independent macropinocytosis. Ultimately, L-K6 causes cancer cell death through nuclear targeting and DNA damage, without significant cell membrane, cytoskeleton and mitochondria disruptions. All these findings may provide a clear schemetic illustration of how L-K6 interacts with MCF-7 cancer cells. The data of present study may also help understand the complicated interactions between CAPs and cancer cells.

## Materials and Methods

### Cell lines and culture

Eleven human cancer cell lines (including MCF-7 breast carcinoma, Bcap-37 breast carcinoma, MDA-MB-231 breast adenocarcinoma, HeLa cervical carcinoma, BGC-823 gastric carcinoma, A375 melanoma, NCI-H446 small cell lung carcinoma, HO-8910 ovarian carcinoma, SMMC-7721 hepatocellular carcinoma, LK-2 lung squamous cells carcinoma, and BEL-7402 hepatocellular carcinoma) were obtained from the CBCAS (Cell Bank of the Chinese Academy of Sciences, Shanghai, China). Human HaCaT keratinocytes were obtained from KeyGEN Biotech (Nanjing, China). Cells were routinely cultured in RPMI 1640 or L15 or DMEM containing 10% neonatal bovine serum (NBS) or fetal bovine serum (FBS), 1% l-glutamine, 1% sodium pyruvate, 50 U/mL penicillin and 50 μg/mL streptomycin. The cells were maintained at 37 °C in a humidified atmosphere of 5% CO_2_.

### Peptides sequences and synthesis

Peptides (L-K6, L-K5V1, L-K6V1, Table 1) were synthesized in crude form by the standard Fmoc solid-phase peptide synthesis protocols of GL Biochem Ltd. (Shanghai, China) and were purified to near homogeneity (>95%) by reverse-phase HPLC. The relative masses of peptides were determined by using HPLC-MS and MALDI-TOF MS (Shimadzu, Japan).

### Cell viability detection by MTT assay

Cell viability was detected by MTT assay. The cells were seeded at 5 × 10^3^ cells/well in 96-well plates 24 h before the peptide treatment. After 1, 6, 24, or 48 h of incubation with various concentrations of peptides (20–100 µM), 10 μL of the 5 mg/mL MTT solution was added to each well and incubated for additional 4 h. The purple-blue MTT formazan precipitate was dissolved in 150 μL of DMSO. The absorbance was determined using a microplate reader at 490 nm. The experiments were performed in triplicate. The data were expressed as a percentage of the inhibition rate for the viable cells. For the thermo-sensitivity test, MCF-7 cells were treated with L-K6 at 4 °C or 37 °C for 30 min and then subjected to the MTT assay. For energy-dependent assay, the L-K6-treated MCF-7 cells were pre-incubated with 40 μM NaN_3_ for 30 min^39^ and then subjected to the MTT assay. All experiments had been performed in triplicate.

### Hemolysis assay

Fresh human erythrocytes were collected by centrifugation for 10 min at 1,500 × g, and washed three times with normal saline (NS), then diluted in NS to a concentration of 2%. The erythrocytes were added to each well and incubated at 37 °C with various concentrations of peptides (0–400 μM) for 1 hour. The release of hemoglobin was monitored by measuring the absorbance of supernatant at 540 nm. Erythrocytes in NS and distilled water were used as control of 0 and 100% hemolysis, respectively. The hemolysis percentage was calculated using the following equation: hemolysis (%) = (A − A0)/(Ax − A0) × 100, where A is OD540 nm with peptide solution; A0 is OD540 nm with NS; Ax is OD540 nm with distilled water.

### Membrane depolarization assay

To evaluate possible membrane permeabilization after L-K6 peptide treatment, 2 µM of bis-(1,3-dibutylbarbituric acid) trimethin eoxonol [DiBAC_4_(3)] was co-incubated with cells for 10 min at 37 °C. Cells were subjected to time scanning using a fluorescence spectrophotometer (Ex 488 nm/Em 518 nm). When the fluorescence intensity was stable, the cells were treated with various concentrations of L-K6. Membrane depolarization was monitored by recording the changes in the fluorescent intensity of DiBAC_4_(3).

### Calcein AM and ethidium homodimer (EthD-1) staining

The cell membrane permeability was evaluated using a two-color fluorescence assay with EthD-1 and calcein AM. MCF-7 cells or HaCaT cells were seeded into 96-well plates at 5 × 10^3^ cells/well. After exposure to various concentrations of L-K6 for 1 hour, the medium was removed, and 20 µL of dye containing 2 µM calcein AM or 4 µM EthD-1 was added and incubated for 30 min in the dark. The fluorescence intensity was determined by fluorescence activated cell sorting (FACs) analysis with an excitation wavelength (Ex) of 485 nm and an emission wavelength (Em) of 530 nm for calcein and Ex 530 nm and Em 645 nm for EthD-1.

### Morphological observation by electronic microscopy

To assess the possible impacts of L-K6 on MCF-7 cancer cells or HaCaT cells, in the present study, the morphological changes of MCF-7 or HaCaT cells were observed after 1 hour peptide treatment by scanning electronic microscopy (SEM, KYKY-1000B, China) and transmission electron microscope (TEM, JEM-200EX, Japan) using standard protocols.

### Live-cell imaging by confocal microscopy

To assess the preferential impacts of L-K6 on MCF-7 cancer cells surface, in the present study, live-cell imaging was performed by confocal fluorescent microscopy to track the dynamic morphological changes of the cell surface after peptide exposure. MCF-7 and HaCaT cells (1 × 10^6^ cells) were seeded in glass bottom culture dishes and left to adhere to the covers-lips for >12 h. Cells were incubated at 37 °C in complete medium (1 mL) containing 50 μM L-K6. Before visualization, medium containing peptides was removed from the cells by aspiration, and the cells were washed three times with fresh medium (37 °C). Finally, clear complete media was added and cells were subsequently viewed for a maximum of 30 min by using confocal microscopy (LSM710, Zeiss, Germany).

### Cellular uptake of L-K6 by MCF-7 cells as assessed by confocal microscopy

MCF-7 or HaCaT cells were incubated with various concentrations of FITC-labeled L-K6 for 5, 10 or 60 min. Myelin and other lipophilic areas on cell membrane were stained with the red-orange fluorescent tracker DiI. The FITC-labeled peptides were traced and recorded at each time point using confocal microscopy.

### Assessment of cellular uptake of L-K6 by using super-resolution microscopy

MCF-7 cells were treated with FITC-labeled L-K6 (10–50 μM) in the presence or absence of various endocytosis inhibitors, cytochalasin D, ethylisopropylamiloride, methyl-β-cyclodextrin, or chlorpromazine, for 1 hour. The cellular uptake of peptides was tracked using super-resolution microscopy (Deltavision OMX, GE, USA).

### Assessment of cellular uptake of L-K6 by using FACs analysis and microplate reader

After 20 min pretreatment of MCF-7 cells with various endocytosis inhibitors, the culture media was removed, washed by PBS and added medium containing FITC-labeled L-K6 (final concentrations 10–50 μM) for 1 hour. The cellular uptake of peptides was determined by using FACs analysis. For microplate reader analysis, the peptides-treated MCF-7 cells were disintegrated by 5% Triton X-100 in 0.2 N NaOH, and the fluorescence was determined by using a Fluorescence Microplate Reader.

### Circular dichroism spectroscopy measurements

The circular dichroism spectra of peptides was performed using spectropolarimeter (Bio-Logic, MOS-500, France) under nitrogen flush in 1 mm path length cell at 25 °C. The analysis was performed under indicated conditions (50% trifluoroethanol, 30% SDS, water or 0.8 mM liposome). Liposome was composed of phosphatidylcholine (PC), PS and cholesterol (CHO) with PC/PS/CHO ratio (mol/mol) at 9:1:1, 7:3:1 or 5:5:1. The spectra were recorded between 190 and 250 nm. The percentage of the α-helical structure was calculated by CDPro software package.

### Fluorescence dyes leakage from liposome vesicles

In order to detect potential roles of PS on the interactions between L-K6 and MCF-7 cell sueface, phospholipid liposomes composed of PC, PS and CHO were prepared using the reverse-phase evaporation. Phospholipid liposomes containing 9:1:1, 7:3:1 or 5:5:1 molar ratios of PC/PS/CHO were constructed. Calcein, one fluorescence dye (molecular weight, 623 Da; diameter, ~1 nm) was encapsulated into liposome vesicles. Liposome vesicles were seeded onto 96-well microplates and coincubated with L-K6 for 1 hour. The fluorescence leakage was detected under 485 nm and 520 nm excitation and emission wavelengths, respectively. All experiments had been performed in triplicate.

### Isothermal titration calorimetry (ITC) assay

In order to confirm the direct binding of L-K6 to PS, ITC analysis was performed at 30 °C on a Microcal high-sensitivity ITC calorimeter (ITC-200, GE, USA). Liposomes of 1.5 mM PS/Cholesterol were injected into the chamber containing 1.0 mM L-K6. L-K6 peptides were titrated with liposomes as control. Each injection of 2 μL was done in a 5-s period with 120-s interval. The heats of dilution were determined in control experiments by injecting lipid vesicles into buffer solutions and subtracting the heat produced from the corresponding peptide-PS binding experiment.

### FACS analysis of mitochondrial membrane potential, intracellular calcium (Ca^2+^) and reactive oxygen spedies (ROS) production

The mitochondrial membrane potential was measured using rhodamine 123 (Rhd-123) fluorescence. One hour after L-K6 exposure, the cells were loaded with 10 µM Rho-123 and incubated at 37 °C for 30 min in the dark. The cells were then harvested, washed, resuspended in PBS and immediately analyzed using flow cytometry with excitation and emission wavelengths of 485 nm and 520 nm, respectively.

To detect the cytosolic Ca^2+^ level, cells were incubated with 4 μM Ca^2+^-sensitive fluorescent Fluo-3-AM in Hank’s buffered salt solution (HBSS) with 1.3 mM Ca^2+^ for 30 min at 37 °C. The cells were then washed twice with fresh HBSS and incubated in HBSS at room temperature prior to detection. The green fluorescence from Fluo-3-AM (Ex 488 nm/Em 526 nm) was proportional to the cytosolic free Ca^2+^ concentrations.

The intracellular ROS production was determined using 2′,7′-dichlorofluorescin- diacetate (DCFH-DA), a sensitive free-radical indicator which can be oxidized by ROS to form a green fluorescent molecule, 2′,7′-dichlorofluorescein (DCF). The L-K6-treated MCF-7 cells were incubated with 25 µM DCFH-DA for 30 min in the dark. After the incubation, the cells were collected, washed with PBS, resuspended in PBS and subjected to flow cytometry analysis. All experiments had been performed in triplicate.

### Electrophoretic mobility shift assay (EMSA)

To test the interactions of L-K6 to DNA, the bacteria genomic DNA and MCF-7 cell genomic DNA were extracted and EMSA assay was performed. The bacterial genomic DNA was mixed with peptides for 30 min with peptide/DNA mass ratios at 0:1, 0.5:1, 1:1, 5:1, 10:1, 50:1 and 100:1, respectively. After adding 3 μL of loading buffer (Takara, Dalian, China), the DNA and peptide complex was resolved by 1% agarose gel electrophoresis and the migrated DNA was visualized using ethidium bromide (EB) fluorescence under UV light.

### Competitive binding of L-K6 and EB with genomic DNA

The cellular DNA was dissolved in 1 mL of TE buffer (10 mM, pH8.0), then 15 μL of EB solution was added. The mixture was incubated for 10 min at 37 °C in the dark. L-K6 (10, 30 and 50 μM) was then added to the EB–DNA mixture and fluorescence spectra were measured after 1 hour of incubation at 37 °C in the dark. The solutions were excited at 535 nm, and the spectra were recorded from 550 to 750 nm.

### DAPI nuclear staining assay

The peptide-treated cells were collected and washed with PBS followed by fixation with 2% paraformaldehyde at 4 °C for 30 min. The cells were stained with DAPI (0.5 μg/mL) for 30 min, and then washed and mounted on slides using a cytospinner. The nuclear morphology was visualized by inverted fluorescence microscope.

### Super-resolution microscopy observation of the cytoskeleton and nucleus

The MCF-7 or HaCaT cells were co-incubated with L-K6 (10, 30 and 50 μM) for 1 hour, and then their cytoskeleton or nucleus were labeled with anti-α-tubulin/Alexa Fluor 488 or Hoechst 33342 and observed using super-resolution microscopy.

### *In vivo* assessment of the anticancer activity of L-K6 using a xenograft model

All experiments procedures were performed in accordance with the Laboratory Animal Care and Use Guidelines approved by the Animal Care and Use Committee of Liaoning Normal University. Female nude mice at the age of 6 to 8 weeks were obtained from Vital River Laboratories (Beijing, China). Approximately 1 × 10^7^ MCF-7 cells were suspended in 100 μL NS and subcutaneously injected into the right flank of the mice. At the onset of a palpable tumor (approximately 100 mm^3^), 15 mice were divided into 3 groups and were locally injected with 5 or 10 mg/kg L-K6, or an equivalent volume NS every day for 15 days. The tumor size was measured everyday. At the end of the experiment, all animals were sacrificed and the tumor masses were weighed. The pathological analysis was performed by H&E staining.

### Statistical Analysis

All the data were presented as mean ± standard deviation. Statistical analysis (ANOVA and Tukey’s posthoc analysis) was performed by using SPSS version 14.0. A p value of < 0.05 showed significant difference between groups.

## Electronic supplementary material

Supplementary Information 
