# Supplementary material for: Cell surface binding, uptaking and anticancer activity of L-K6, a lysine/leucine-rich peptide, on human breast cancer MCF-7 cells
Source: Sci Rep. 2017 Aug 15;7:8293. doi: 10.1038/s41598-017-08963-2 (PMC5557901; doi:10.1038/s41598-017-08963-2)
Supplement: Supplementary file 1 — Supplementary Information [file 41598_2017_8963_MOESM1_ESM.pdf]

Supplementary Info

Cell surface binding, uptaking and anticancer activity of L-K6, a lysine/leucine-rich peptide, on human breast cancer MCF-7 cells

Che Wang [1,2], Shaodan Dong [1], Lin Zhang [1], Ying Zhao [1], Lili Huang [2], Xiange Gong [2], He Wang [2], Dejing Shang [2]*

[1] Department of Pharmacy, School of Chemistry and Chemical Engineering, Liaoning Normal University, Dalian 116029, China;

[2] Liaoning Provincial Key Laboratory of Biotechnology and Drug Discovery, School of Life Sciences, Liaoning Normal University, Dalian 116081, China.

Supplementary Figures Legends

Suppl Fig. S1. Effects of endocytosis inhibitors on the cellular uptake of FITC-labeled L-K6 peptides in MCF-7 cells, as assessed by flow cytometry and a microplate reader. Consistent with the super-resolution microscopy observation, the FACS (a) and microplate reader (b) data also revealed that MβCD, EIPA and CyD could reduce the internalization of L-K6 into MCF-7 cells, whereas CPZ did not ameliorate the cellular uptake of L-K6. Additionally, this interrupted uptake of L-K6 by the various endocytosis inhibitors leads to reduced cytotoxicity (c,d). Additionally, the microplate data also indicated that low temperature (4 ˚C) will decrease L-K6 uptake (b, right panel), which was consistent with the reduced cytotoxicity of L-K6 at 4 ˚C (Fig. 1c)

Suppl Fig. S2. FACS analysis of the mitochondrial membrane potential using Rhodamine123 (a), intracellular calcium (Ca^{2+}) levels using Fluo-3 AM (b) and ROS production using DCFH-DA (c), after a one-hour L-K6 exposure to MCF-7 cells. The data indicated that after one hour of treatment, L-K6 induced only a slight reduction of the mitochondrial membrane potential (a). The cytosolic Ca^{2+} level was also slightly decreased at higher concentrations (b), which may be due to the membrane damage and the elevated membrane permeability. In addition, ROS production was also elevated after one hour of L-K6 exposure (c).

Suppl Fig. S3. Control experiment of ITC test.

Suppl Fig. S4. Schemetic illustration of cellular process and cytotoxic mechanisms of L-K6 in MCF-7 cells.

a

	Cellular uptake of L-K6 (% of L-K6-treated cells)		
	10 μM	30 μM	50 μM
L-K6 + CyD	16.1 ± 2.3 % **	6.4 ± 1.1 % **	47.8 ± 4.9 % **
L-K6 + Mβ CD	43.5 ± 5.1 % **	63.8 ± 5.4 % *	82.3 ± 7.2 %
L-K6 + EIPA	89.5 ± 9.6 %	89.9 ± 6.4 %	63.6 ± 5.7 % *
L-K6 + CPZ	107.5 ± 12.1 %	135.5 ± 7.7 % *	94.2 ± 5.5 %

b

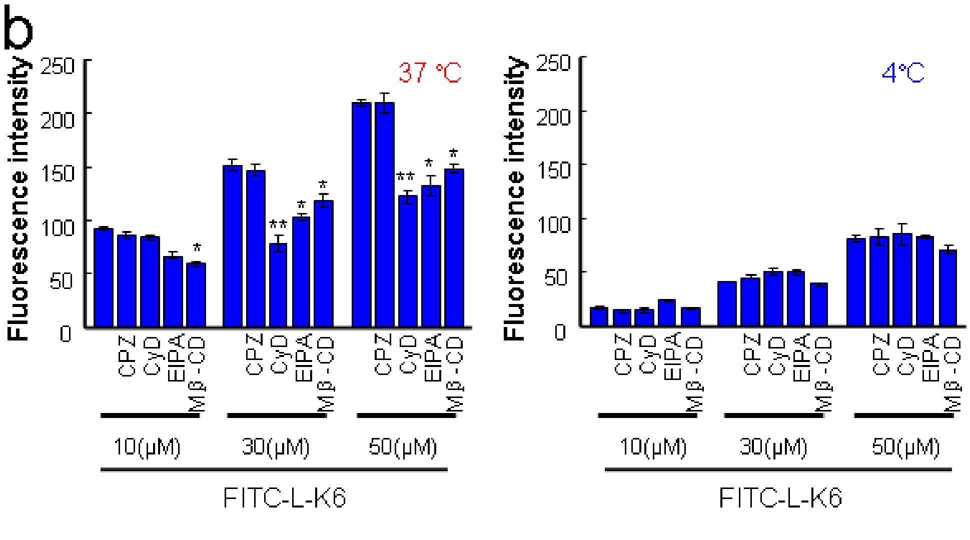

c

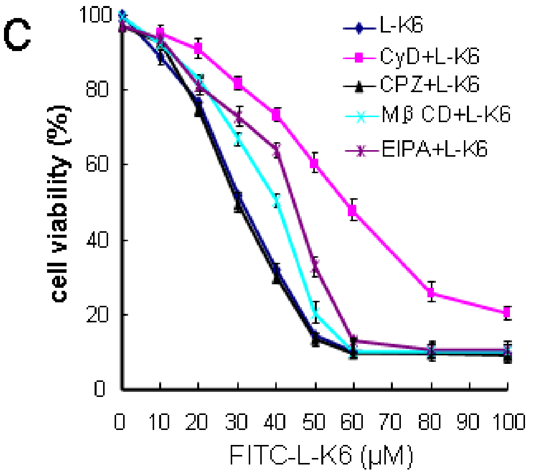

d

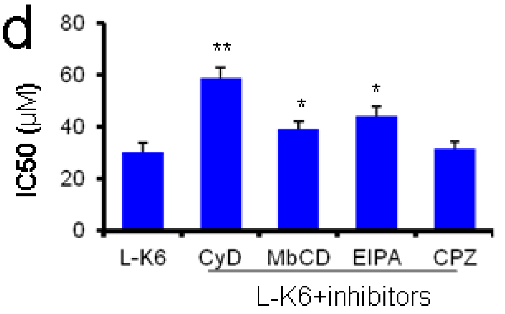

Fig. S1

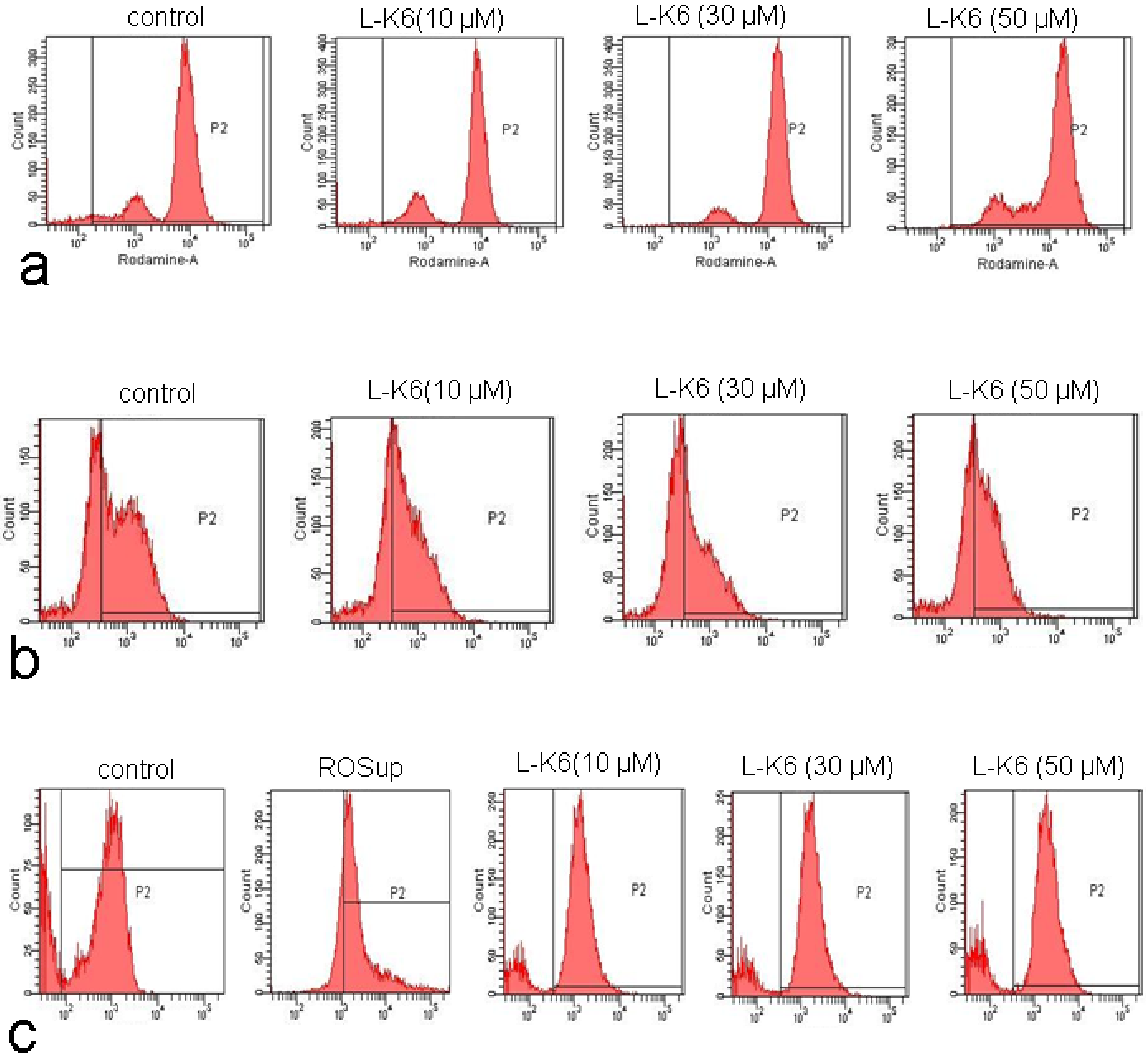

control
L-K6(10 μM)
L-K6 (30 μM)
L-K6 (50 μM)
Count
P2
Rodamine-A
a
control
L-K6(10 μM)
L-K6 (30 μM)
L-K6 (50 μM)
Count
P2
b
control
ROSup
L-K6(10 μM)
L-K6 (30 μM)
L-K6 (50 μM)
Count
P2
c

Fig. S2

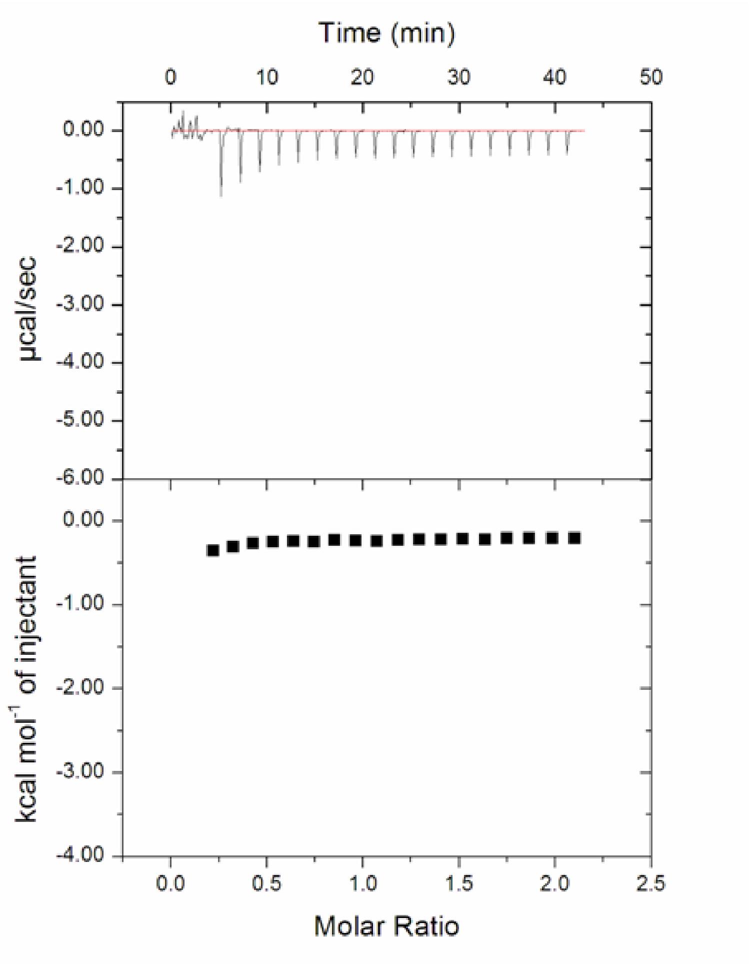
Time (min)
0 10 20 30 40 50
0.00
-1.00
-2.00
-3.00
-4.00
-5.00
-6.00
µcal/sec
0.00
-1.00
-2.00
-3.00
-4.00
kcal mol-1 of injectant
0.0 0.5 1.0 1.5 2.0 2.5
Molar Ratio

Fig. S3

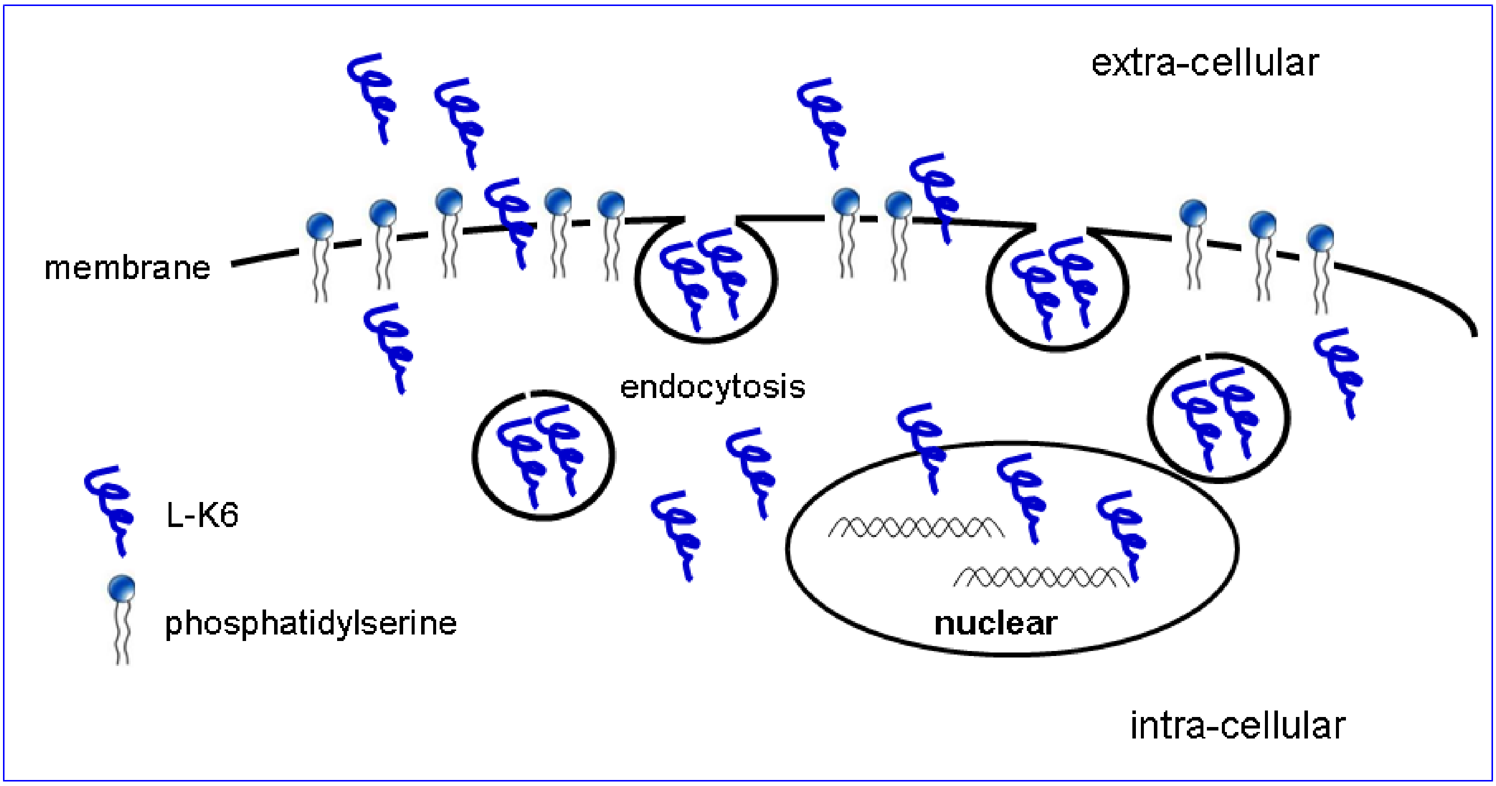
extra-cellular
membrane
endocytosis
L-K6
phosphatidylserine
nuclear
intra-cellular

Fig. S4
